# Utilization of a Wheat Sidestream for 5-Aminovalerate Production in *Corynebacterium glutamicum*

**DOI:** 10.3389/fbioe.2021.732271

**Published:** 2021-09-29

**Authors:** Arthur Burgardt, Carina Prell, Volker F. Wendisch

**Affiliations:** Genetics of Prokaryotes, Faculty of Biology and CeBiTec, Bielefeld University, Bielefeld, Germany

**Keywords:** *Corynebacterium glutamicum*, wheat sidestream concentrate, hydrolysates, flux enforcement, 5-aminovalerate

## Abstract

Production of plastics from petroleum-based raw materials extensively contributes to global pollution and CO_2_ emissions. Biotechnological production of functionalized monomers can reduce the environmental impact, in particular when using industrial sidestreams as feedstocks. *Corynebacterium glutamicum*, which is used in the million-ton-scale amino acid production, has been engineered for sustainable production of polyamide monomers. In this study, wheat sidestream concentrate (WSC) from industrial starch production was utilized for production of l-lysine–derived bifunctional monomers using metabolically engineered *C. glutamicum* strains. Growth of *C. glutamicum* on WSC was observed and could be improved by hydrolysis of WSC. By heterologous expression of the genes *xylA*_*Xc*_ *B*_*Cg*_ (*xylA* from *Xanthomonas campestris*) and *araBAD*_*Ec*_ from *E. coli*, xylose, and arabinose in WSC hydrolysate (WSCH), in addition to glucose, could be consumed, and production of l-lysine could be increased. WSCH-based production of cadaverine and 5-aminovalerate (5AVA) was enabled. To this end, the lysine decarboxylase gene *ldcC*_*Ec*_ from *E. coli* was expressed alone or for conversion to 5AVA cascaded either with putrescine transaminase and dehydrogenase genes *patDA*_*Ec*_ from *E. coli* or with putrescine oxidase gene *puo*_*Rq*_ from *Rhodococcus qingshengii* and *patD*_*Ec*_. Deletion of the l-glutamate dehydrogenase–encoding gene *gdh* reduced formation of l-glutamate as a side product for strains with either of the cascades. Since the former cascade (*ldcC*_*Ec*_-*patDA*_*Ec*_) yields l-glutamate, 5AVA production is coupled to growth by flux enforcement resulting in the highest 5AVA titer obtained with WSCH-based media.

## Introduction

The production of plastics from petroleum-based raw materials extensively contributes to global pollution and CO_2_ emissions. While bio-based polymers from crops produce significantly lower carbon emissions and have lower energy production requirements (Meereboer et al., 2020), their scalability is limited by competition with food industries, acreage of land, and consumption of water and nutrients (Chia et al., 2020). Additionally, climate change severely affects the agricultural industry by extreme weather anomalies (Beniston et al., 2007); in particular, the cultivation of crops such as grains and maize, as well as vegetable and fruit production was affected. Consequently, it is essential for a biotechnological industry to use agricultural feedstocks more efficiently. One main strategy is the shift toward feedstocks that are not competitive with human and animal nutrition. This includes the use of industrial sidestreams as well as lignocellulose-derived sugars like xylose and arabinose (Wendisch et al., 2016). Most industrial platform strains are not able to naturally utilize these nonfood feedstocks or produce value-added compounds in high titers in their wild-type form (Wendisch et al., 2016). Metabolic engineering and systems biology were indispensable to establish microbial cell factories independent from glucose (Dai and Nielsen, 2015; Erb et al., 2017). In recent years, the model organism *C. glutamicum* was extensively engineered to broaden its substrate spectrum according to the flexible feedstock concept (Wendisch et al., 2016). Thereby, access to the lignocellulosic pentoses arabinose (Kawaguchi et al., 2008; Schneider et al., 2011; Meiswinkel et al., 2013) and xylose (Kawaguchi et al., 2006; Jin et al., 2020) for growth and production of amino acids was enabled. Recently, it could also be demonstrated that production of sarcosine from the pentose xylose was more efficient than that from glucose (Mindt et al., 2019b). Additionally, less processed substrates such as lignocellulosic residuals, rich straw, wheat bran and plant mass hydrolysates, chitin, the biorefinery sidestream pyrolysis water, and wheat sidestream concentrate (WSC) from the starch and paper industries could be harnessed by *C. glutamicum* as carbon sources (Gopinath et al., 2011; Lange et al., 2017; Mindt et al., 2019a; Sasaki et al., 2019; Prell et al., 2021a; Vortmann et al., 2021).

A special focus lies on the fermentative production of amino acids by *C. glutamicum*, which has been well established over the past decades (Wendisch, 2020). In general, amino acids have a wide spectrum of applications in the food, feed, and pharmaceutical industries. The annual production of l-glutamate and l-lysine reached 3,210,000 and 2,600,000 metric tons, respectively (Wendisch, 2020). The production of l-lysine from alternative carbon sources has been successfully established from whey (Barrett et al., 2004) and starch (Seibold et al., 2006). The high industrial impact of the amino acid production by *C. glutamicum* formed the basis for the synthesis of amino acid–based derivatives (Pérez-García et al., 2017, 2018; Mindt et al., 2018; Veldmann et al., 2019). Some prominent derivatives of the amino acid l-lysine are the bifunctional monomers cadaverine, 5-aminovalerate (5AVA), and glutarate, which are monomeric precursors for the production of bio-polyamides. As plastics are primarily synthesized from natural gas and petroleum, the worldwide interest in bio-based production of the functional monomers is steadily increasing. Polyamides can be either obtained by anionic ring-opening polymerization of lactams, the cyclization products of ω-amino acids, or by condensation of dicarboxylic acids with diamines (Radzik et al., 2020). The production of these monomeric building blocks for polyamides has been established in metabolically engineered *C. glutamicum* and *E. coli* (Chae et al., 2020; Wendisch, 2020). Diamines like putrescine (Qian et al., 2009; Schneider et al., 2012) and cadaverine (Na et al., 2013; Kind et al., 2014) as well as the ω-amino acids γ-4-aminobutyrate (GABA) (Choi et al., 2015; Jorge et al., 2017a) and 5AVA (Jorge et al., 2017b; Haupka et al., 2020), and the dicarboxylic acids succinate and glutarate (Okino et al., 2008; Pérez-García et al., 2018; Han et al., 2020) were successfully produced in high titers.

The C5-ω-amino acid 5AVA, the monomeric precursor of the polyamide 5 (PA 5) (Chae et al., 2017; Zhang et al., 2017), can be derived from l-lysine by four different (synthetic) pathways. In the first pathway, l-lysine is converted to 5AVA by l-lysine-α-oxidase (RaiP) from *Scomber japonicas* by oxidative deamination and a spontaneous decarboxylation step (Cheng et al., 2020). Recently, an alternative synthetic route was established starting with RaiP, but the intermediate 2-keto-6-aminocaproate is converted by α-ketoacid decarboxylase (KivD) from *Lactococcus lactis* and aldehyde dehydrogenase (PadA) from *Escherichia coli* to 5AVA (Cheng et al., 2021). The second pathway to 5AVA combines oxidative decarboxylation by l-lysine monooxygenase (DavA) using molecular oxygen followed by desamidation by γ-aminovaleramidase (DavB) from *P. putida* (Adkins et al., 2013). The third and the fourth pathways are both initiated by decarboxylation of l-lysine catalyzed by l-lysine decarboxylase (LdcC) from *E. coli* (Jorge et al., 2017b; Haupka et al., 2020). The third pathway employs putrescine oxidase Puo from *Rhodococcus qingshengii*, which requires molecular oxygen and NAD-dependent γ-aminobutyraldehyde dehydrogenase PatD from *E. coli* for conversion of cadaverine to 5AVA (Haupka et al., 2020). The fourth pathway does not require molecular oxygen and converts cadaverine to 5AVA through 2-oxoglutarate-dependent putrescine/cadaverine transaminases PatA and PatD from *E. coli* (Jorge et al., 2017b). Later, this pathway was also used with alternative enzymes, namely l-lysine decarboxylase CadA from *E. coli*, 2-oxoglurate-dependent putrescine transaminase KpcPA and NAD-dependent γ-aminobutyraldehyde dehydrogenase KpcPD from *Klebsiella pneumoniae* (Wang et al., 2021). The transaminase reaction, e.g., in the LdcC-PatA-PatD cascade, yields l-glutamate from 2-oxoglutarate. Flux enforcement in a metabolic setup in which growth requires production of 5AVA can be achieved by deletion of *gdh*, the gene coding for the major ammonium assimilating enzyme l-glutamate dehydrogenase as shown for glutarate production (Pérez-García et al., 2018; Haupka et al., 2020). In a comparison of two cascades with either one (LdcC-PatA-PatD-GabT-GabD) or two transaminase reactions (LdcC-Puo-PatD-GabT-GabD), a 1:1 stoichiometry proved to be superior to coupling with two transaminases providing l-glutamate (Pérez-García et al., 2018; Haupka et al., 2020).

**FIGURE 1:**
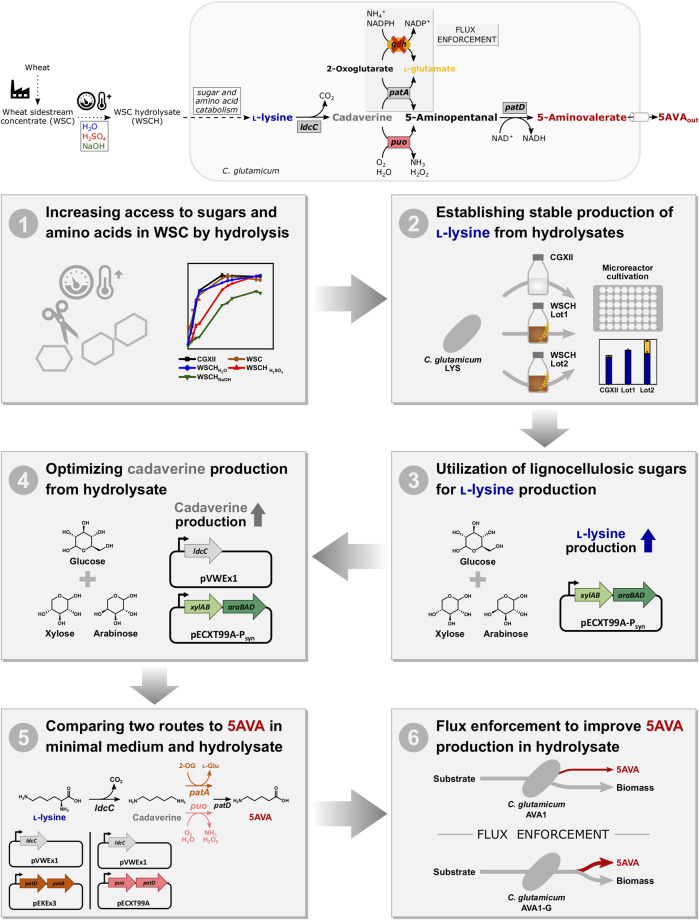
Schematic overview of the metabolic route and workflow from the feedstock WSCH to the product 5AVA. *ldcC*, L-lysine decarboxylase; *gdh*, L-glutamic acid dehydrogenase; *patA*, putrescine transaminase; *puo*, putrescine oxidase; *patD*, γ-aminobutyraldehyde dehydrogenase; *xylA*, xylose isomerase; *xylB*, xylulose kinase; *araB*, ribulokinase; *araA*, L-arabinose isomerase; *araD*, L-ribulose-5-phosphate 4-epimerase; 2-OG, 2-oxoglutarate; L-Glu, L-glutamate; and 5AVA, 5-aminovalerate.

To produce bio-based polyamides from sustainable resources, *C. glutamicum* was engineered to produce cadaverine from starch (Tateno et al., 2007, 2009) and from xylose (Buschke et al., 2013). Also, other model organisms like *E. coli* have been successfully engineered, and cadaverine could be produced from soybean hydrolysates (Guo et al., 2021). Aside from cadaverine, ω-amino acids GABA and 5AVA were produced in *C. glutamicum* from empty fruit bunch sugars (Baritugo et al., 2018) and from a *Miscanthus* and a rice straw hydrolysate (Joo et al., 2017; Sasikumar et al., 2021), respectively. As hydrolysates are mainly obtained directly from grains, a new perspective would be the processing of sidestreams from industrial processes. It was shown before that WSC from the paper industry can be applied as a medium for the production of the dicarboxylic acid glutarate and the trifunctional molecule l-2-hydroxyglutarate (Prell et al., 2021a). In this study, the WSC and different hydrolysates derived from it were investigated for production of l-lysine and its derivatives cadaverine and 5AVA (Figure 1).

## Materials and Methods

### Microorganisms and Cultivation Conditions

*C. glutamicum* ATCC 13032 derived strains were cultivated in brain heart infusion with 0.5 M sorbitol (BHIS), supplemented with 25 μg mL^−1^ kanamycin, 100 μg mL^−1^ spectinomycin, and 5 μg mL^−1^ tetracycline, if necessary. All bacterial strains and plasmids are listed in Tables 1, 2.

**TABLE 1:** Bacterial strains used in this study.

Strain	Relevant Characteristics	References
*E. coli* DH5α	∆*lac*U169 (φ80*lac*Z ∆M15), *sup*E44, *hsd*R17, *rec*A1, *end*A1, *gyr*A96, *thi*-1, and *rel*A1	Hanahan, (1985)
*E. coli* S17-1	*recA*, *pro*, *hsdR*, RP4- 2Tc∷Mu Km∷Tn7 integrated into the chromosome	Simon et al. (1983)
*C. glutamicum* GRLys1 (DM1933ΔCGP123)	*C. glutamicum* ATCC 13032 with modifications: Δ*pck*, *pyc* ^P458S^, hom^V59A^, 2 copies of *lysC* ^T311I^, 2 copies of *asd*, 2 copies of *dapA*, 2 copies of *dapB*, 2 copies of *ddh*, 2 copies of *lysA*, 2 copies of *lysE*, in-frame deletion of prophages CGP1 (cg1507-cg1,524), CGP2 (cg1746-cg1752), and CGP3 (cg1890-cg2071)	Unthan et al. (2014)
GSLA	GRLys1 with in-frame deletions: *sugR* (cg2115), *ldhA* (cg3219), and *snaA* (cg1722)	Pérez-García et al. (2018)
GSLA2	GSLA with in-frame deletion: *cgmA* (cg2893)	Pérez-García et al. (2018)
GSLA2G	GSLA2 with in-frame deletion: *gdh* (cg2280)	Pérez-García et al. (2018)
GSLA2Δ*gabTDP* (=LYS)	GSLA2 with deletion of the *gabTDP* operon (cg0566-cg0568)	Jorge et al. (2017b)
GSLA2GΔ*gabTDP* (=LYS-G)	GSLA2G with deletion of the *gabTDP* operon (cg0566-cg0568)	This study
LYS-XA	GSLA2Δ*gabTDP* (pEC-XT99A-P_syn_-*xylA*_*Xc*_ *B*_*Cg*_ *-araBAD*_*Ec*_)	This study
CAD	GSLA (pVWEx1-*ldcC*_*Ec*_)	This study
CAD-XA	GSLA (pVWEx1-*ldcC*) (pEC-XT99A-P_syn_-*xylA*_*Xc*_ *B*_*Cg*_ *-araBAD*_*Ec*_)	This study
AVA1	GSLA2Δ*gabTDP* (pVWEx1-*ldcC*_*Ec*_) (pEKEx3-*patDA*_*Ec*_)	Jorge et al. (2017b)
AVA1-G	GSLA2GΔ*gabTDP* (pVWEx1-*ldcC*_*Ec*_) (pEKEx3-*patDA*_*Ec*_)	This study
AVA2	GSLA2Δ*gabTDP* (pVWEx1-*ldcC*_*Ec*_) (pEC-XT99A*-puo*_*Rq*_ *-patD*_*Ec*_)	Haupka et al. (2020)
AVA2-G	GSLA2GΔ*gabTDP* (pVWEx1-*ldcC*_*Ec*_) (pEC-XT99A*-puo*_*Rq*_ *-patD*_*Ec*_)	This study

**TABLE 2:** Plasmids used in this study.

Plasmid	Relevant Characteristics	References
pEC-XT99A	Tet^R^, *C. glutamicum*/*E. coli* shuttle vector (P_trc_ *, lacI* ^*q*^, pGA1 o*riV*_*Cg*_)	Kirchner and Tauch, (2003)
pEC-XT99A-*puo*_*Rq*_ *-patD*_*Ec*_	pECXT99A, expressing *puo* from *Rhodococcus qingshengii* and *patD* from *E. coli* MG1655	Haupka et al. (2020)
pEC-XT99A-P_syn_	pEC-XT99A with a synthetic promoter	Henke et al. (2021)
pEC-XT99A-P_syn_- *xylA*_*Xc*_ *B*_*Cg*_	pEC-XT99A, expressing *xylA* from *Xanthomonas campestris* and *xylB* from *C. glutamicum* under a synthetic promoter	Henke et al. (2021)
pEC-XT99A-P_syn_- *xylA*_*Xc*_ *B*_*Cg*_ *-araBAD*_*Ec*_	pEC-XT99A, expressing *xylA* from *Xanthomonas campestris, xylB* from *C. glutamicum* and *araBAD* from *E. coli* under a synthetic promoter	This study
pEKEx3	Spec^R^, *C. glutamicum*/*E. coli* shuttle vector (P_tac_ *lacI* ^*q*^ pBL1, *oriV*_*Ec*_)	Stansen et al. (2005)
pEKEx3-*patDA*_*Ec*_	pEKEx3, expressing *patD* and *patA* from *E. coli* MG1655	Jorge et al. (2017b)
pVWEx1	Kan^R^, *C. glutamicum*/*E. coli* shuttle vector (P_tac_, *lacI* ^*q*^)	Peters-Wendisch et al. (2001)
pVWEx1-*araBAD*_*Ec*_	pVWEx1 expressing *araBAD* from *E. coli* MG1655	Schneider et al. (2011)
pVWEx1-*ldcC*_*Ec*_	pVWEx1 expressing *ldcC* from *E. coli* MG1655	Jorge et al. (2016)
pK19*mobsacB*	Kan^R^, mobilizable *E. coli* vector mutagenesis (*oriV*, *sacB*)	Schäfer et al. (1994)
pK19*mobsacB*-Δ*gdh*	pK19*mobsacB* from *C. glutamicum* with a construct for deletion of *gdh* (cg2280)	Pérez-García et al. (2018)

Growth experiments with *C. glutamicum* ATCC 13032 in WSC and the different hydrolysates were performed in 10 mL Duetz microcultivation plates (Kuhner Shaker GmbH, Herzogenrath, Germany) with culture volumes of 3 or 2 mL at 220 rpm in an Ecotron ET25-TA-RC (Infors HT, Einsbach, Germany), and plate sandwich covers for low evaporation (1.2 mm hole diameter) were used. Growth was monitored by determination of the OD_600_ with a V-1200 Spectrophotometer (VWR, Radnor, PA, United States). As controls, *C. glutamicum* ATCC 13032 was cultivated in CGXII minimal medium, supplemented with 20 g L^−1^ glucose, and in WSC medium, consisting of 112 g L^−1^ WSC dry weight, 20 g L^−1^ ammonium sulfate as the nitrogen source and 42 g L^−1^ 3-(*N*-morpholino)propanesulfonic acid (MOPS) as buffer (Prell et al., 2021a). For the comparison of different hydrolysates from wheat sidestream concentrate (WSCH), WSCH media contained 80% (*v/v*) hydrolysate (from 140 g L^−1^ WSC), 20 g L^−1^ ammonium sulfate, and 42 g L^−1^ MOPS.

Production experiments with *C. glutamicum* were performed in CGXII minimal medium (Eggeling and Bott, 2005), supplemented with 40 g L^−1^ (220 mM) glucose as a sole carbon source. For comparative l-lysine production in WSCH Lot1 and Lot2, the amount of hydrolysates (from 190 g L^−1^ WSC) was adjusted to a defined glucose concentration of 220 mM (65% (*v/v*) for Lot1 and 74% (*v/v*) for Lot2), and 20 g L^−1^ ammonium sulfate and 42 g L^−1^ MOPS were added. For all remaining production experiments in WSCH, the amount of hydrolysate (from 190 g L^−1^ WSC Lot2) was adjusted to a defined glucose concentration of 190 mM, and 20 g L^−1^ ammonium sulfate and 42 g L^−1^ MOPS were added. If necessary, 1 mM isopropyl-β-d-1-thiogalactopyranoside (IPTG) was added for induction of gene expression.

Overnight cultures in 10 mL BHIS were harvested and washed in TN buffer (50 mM Tris-HCl, 50 mM NaCl, pH 6.3) before inoculation at an initial OD_600_ of 1. The cultivations in the BioLector microcultivation system (m2p-labs, Baesweiler, Germany) were performed in 3.2 mL FlowerPlates at 1,300 rpm with a filling volume of 1,200 μL at 30°C.

### Molecular Biology Methods

Classical methods which include plasmid isolation, molecular cloning, and heat-shock transformation of *E. coli* and electroporation of *C. glutamicum* were performed as described previously (Simon et al., 1983; Eikmanns et al., 1994). ALLin HiFi DNA polymerase (HighQu, Kraichtal, Germany) was used to amplify DNA sequences. For construction of the vector overexpressing *xylA*_*Xc*_ *B*_*Cg*_ *-araBAD*_*Ec*_ under a strong synthetic promoter*, araBAD* was amplified with the primers bx29 (5′-AAC​GCA​GGG​TTG​GTA​CTA​AGA​TCC​TCG​ACA​AGG​AGA​TAT​AGA​TAT​GG-3′) and bx30 (5′-TTG​CAT​GCC​TGC​AGG​TCG​ACT​CTA​GTT​ACT​GCC​CGT​AAT​ATG​CC-3′) using pVWEx1-*araBAD*_*Ec*_ (Schneider et al., 2011) as a template. pEC-XT99A-P_syn_-*xylA*_*Xc*_ *B*_*Cg*_ (Henke et al., 2021) was digested with XbaI and assembled with the amplified DNA using Gibson assembly, yielding pEC-XT99A-P_syn_-*xylA*_*Xc*_ *B*_*Cg*_ *-araBAD*_*Ec*_. The deletion of *gdh* was performed as described previously (Pérez-García et al., 2018).

### Quantification of Amino Acids, Carbohydrates, and Organic Acids by HPLC

The quantification of extracellular amino acids and their derivatives, carbohydrates, and carboxylic acids, in the cultivation medium was performed with a high-performance liquid chromatography system (1200 series, Agilent Technologies Deutschland GmbH, Böblingen, Germany). After centrifugation of 1 mL of cell cultures at 14,000 rpm for 10 min, the supernatant was stored at −20°C prior to analysis. Analysis of l-lysine, 5AVA, and the diamine cadaverine was performed by an automatic pre-column derivatization with *ortho*-phthaldialdehyde (OPA) and separated on a reversed phase HPLC using a pre-column and main column (LiChrospher 100 RP8 EC-5 μ, 125 × 4.6  mm, CS Chromatographie Service GmbH) with l-asparagine as an internal standard (Schneider and Wendisch, 2010). Separation of the amino acids was achieved by a gradient using 0.25% sodium acetate (pH 6.0) and methanol as the mobile phase as described previously (Jorge et al., 2017b). Detection of the fluorescent derivatives was carried out with a fluorescence detector with an excitation wavelength of 230 nm and an emission wavelength of 450 nm. Glucose, xylose, and arabinose concentrations were measured with an amino exchange column (Aminex, 300 × 8 mm, 10 μm particle size, 25 Å pore diameter, CS Chromatographie Service GmbH) under isocratic conditions (5 mM H_2_SO_4_) as described previously with a flow of 0.8 mL min^−1^ (Schneider et al., 2011). The substances were detected with a refractive index detector (RID G1362A, 1200 series, Agilent Technologies) and a diode array detector (DAD G1315B, 1200 series, Agilent Technologies) at 210 nm.

### Hydrolysis of WSC

For preparation of the hydrolysates, 4% (*w/v*) sulphuric acid, 4% (*w/v*) sodium hydroxide, or deionized water was added to 140 or 190 g L^−1^ dry weight of WSC and autoclaved (134°C, 35 min, 1 bar) (Mindt et al., 2019a). The pH was adjusted with potassium hydroxide to 7, followed by centrifugation for 30 min at 4,000 rpm before sterile filtration.

### Reducing Sugar Assay

The reducing sugar content in the WSC and in the WSC-derived hydrolysates was measured as described previously (Gonçalves et al., 2010; Wood et al., 2012).

## Results

### Increased Access to Sugars and Amino Acids by Hydrolysis of Wheat Sidestream Concentrate From the Paper Industry

**FIGURE 2:**
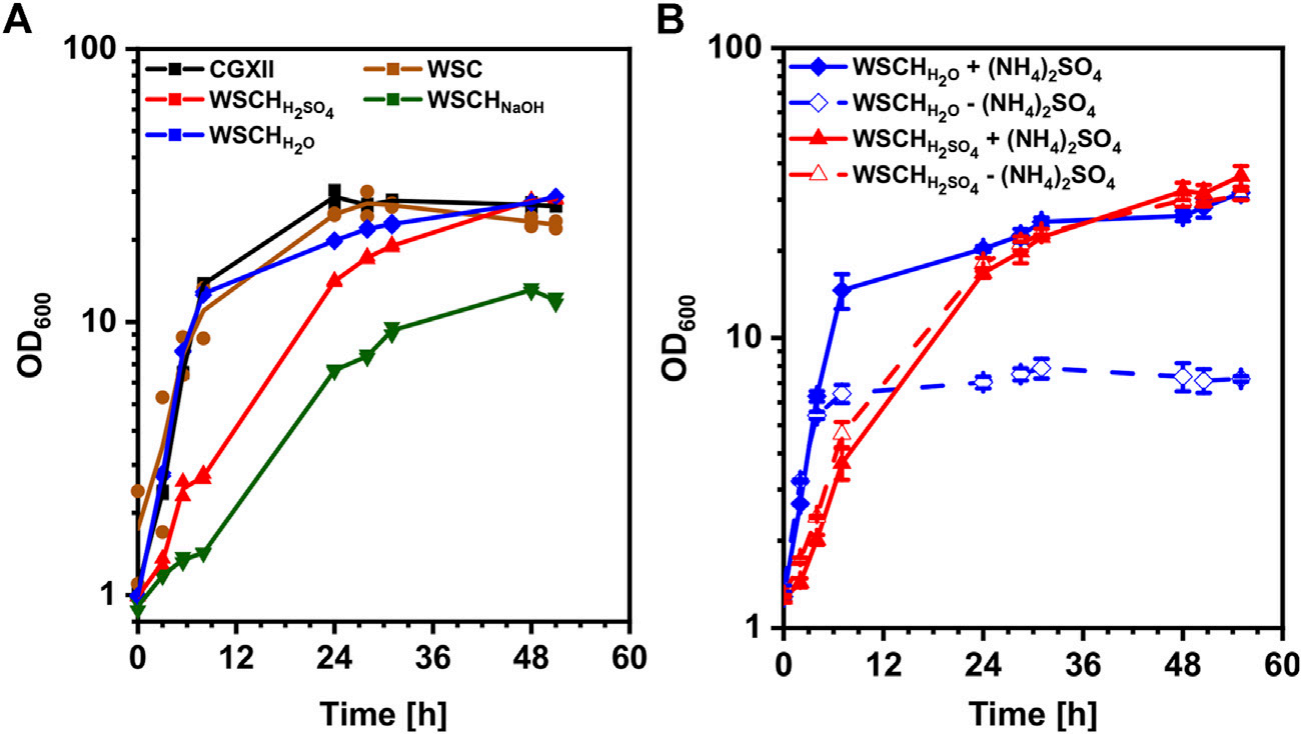
Growth of *C. glutamicum* WT (pVWEx1) in media containing WSC or WSCH. **(A)** Comparison of CGXII, WSC, and its hydrolysates WSCH H_2_SO_4_, WSCH NaOH, and WSCH H_2_O in Duetz plates. Single values are displayed, and lines are drawn as means of duplicates. **(B)** Growth in WSCH_H2SO4_ and WSCH_H2O_ with and without addition of 20 g L^−1^ ammonium sulfate in Duetz plates. Values and error bars represent means and standard deviations (n = of 3 cultivations).

It was tested if a WSC from the starch and paper industry can replace media components including glucose in the commonly used minimal medium CGXII to facilitate growth of *C. glutamicum*. A concentration of 112 g L^−1^ WSC, supplemented with 20 g L^−1^ ammonium sulfate and 42 g L^−1^ MOPS, was tested against CGXII medium with 20 g L^−1^ glucose, resulting in similar growth behavior in both media (Figure 2A). It could be demonstrated that WSC could replace all CGXII media components except the nitrogen source and the buffer (data not shown). However, the finding that the biomass generated from the growth in 112 g L^−1^ WSC did not exceed the biomass from the growth in 20 g L^−1^ glucose suggested that WSC could not be used as efficiently as a carbon source as glucose. To increase access to sugars and amino acids in WSC, acidic, basic, and pH neutral hydrolyses of WSC were performed, and the hydrolysates (WSCH) (80% (*v/v*)) were supplemented with ammonium sulfate and MOPS for growth of *C. glutamicum*. Highest specific growth rates of 0.32 h^−1^ were achieved in CGXII and WSCH_H2O_, followed by WSC (0.23 h^−1^), WSCH_H2SO4_ (0.13 h^−1^), and WSCH_NaOH_ (0.06 h^−1^). In all media except WSCH_NaOH_, an OD_600_ of around 28 was measured, making WSCH_NaOH_ less favorable. Analysis of amino acid and carbohydrate content revealed that compared to WSC, its hydrolysates contained much higher concentrations of amino acids and monosaccharides (Table 3, Supplementary Figures S1, S2, Supplementary Tables S1, S2). Amino acid concentrations in WSCH_H2O_ ranged from 0 to 0.9 mM per 100 g dry mass. In WSCH_H2SO4_, the amino acids aspartate, glutamate, threonine, and glycine stood out with concentrations ranging from 2.9 to 6.0 mM per 100 g dry mass. Main sugars in WSCH_H2O_ were maltose and fructose, whereas in WSCH_H2SO4_, around 184 mM glucose, 38 mM xylose, and 21 mM arabinose per 100 g dry mass were found. When both hydrolysates were used for cultivation with and without addition of ammonium sulfate, no difference was observed for WSCH_H2SO4_. In WSCH_H2O_ without ammonium sulfate, on the other hand, the OD_600_ was capped at 8 (Figure 2B), presumably due to the low amino acid concentrations, compared to WSCH_H2SO4_. Taking all results into consideration, WSCH_H2SO4_ was the preferred hydrolysate and was, therefore, used in all following experiments. For simplicity, WSCH is used as an abbreviation for WSCH_H2SO4_.

**TABLE 3:** Comparison of the different hydrolysates generated from WSC regarding their sugar composition (mM/100 g dry mass) and growth behaviour. Xylose and fructose were quantified as xylose equivalents due to insufficient peak separation.

	WSC	Hydrolysate		
		WSCH_H2O_	WSCH_NaOH_	WSCH_H2SO4_
Reducing sugar content	100.0	269.8	62.7	248.4
Maltose	67.3	60.8	4.4	6.3
Glucose	17.1	23.2	10.5	184.2
Xylose/Fructose	32.4	79.0	16.3	38.3
Arabinose	1.1	5.6	9.6	20.7
Acetate	2.4	4.6	39.7	0.0
µ	0.23 h^−1^	0.32 h^−1^	0.06 h^−1^	0.13 h^−1^
OD_600_	23	29	12	29

### Stable Production of l-lysine From Hydrolysates From Different Batches

**FIGURE 3:**
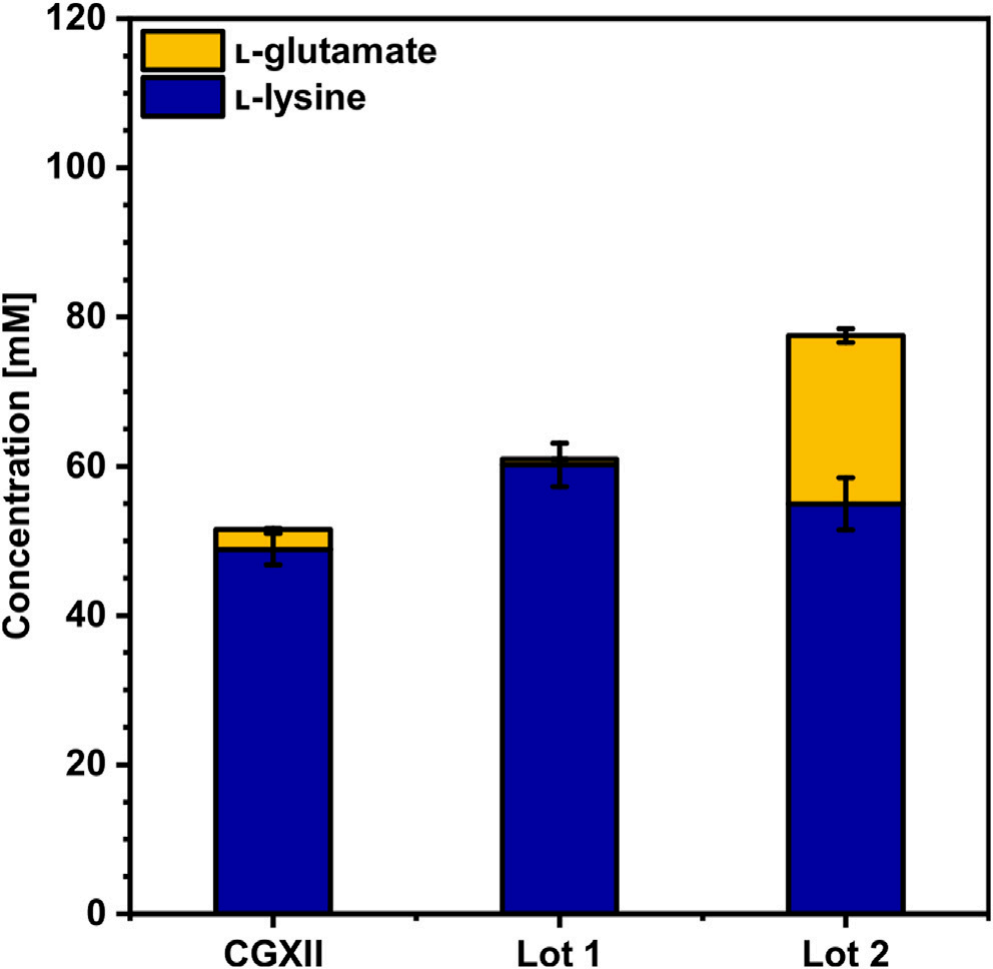
l-Lysine production by *C. glutamicum* LYS in CGXII, WSCH Lot1, and WSCH Lot2, each adjusted to provide 40 g L^−1^ glucose. WSCH was supplemented with 20 g L^−1^ ammonium sulfate and 42 g L^−1^ MOPS. Cultivations were performed in the BioLector microcultivation system. Supernatants were analyzed after 120 h. Values and error bars represent means and standard deviations (n = of 3 cultivations). See Supplementary Table S3 for titers in g L^−1^.

The feed amino acid l-lysine is also the precursor for the synthesis of bifunctional monomers which can be used as building blocks for polyamides. The *C. glutamicum* strain LYS has been engineered to overproduce l-lysine (Jorge et al., 2017b) using CGXII minimal medium with 40 g L^−1^ (220 mM) of glucose. For direct comparison, LYS was cultivated in CGXII and in WSCH hydrolysates prepared from two different batches of WSCH, termed as Lot1 and Lot2, which were adjusted to a glucose concentration of 220 mM (Figure 3, Supplementary Table S3). WSCH was supplemented with MOPS and ammonium sulfate, the latter being necessary to achieve maximal titers. While growth rates in both batches (0.11 and 0.10 h^−1^) were one-third of the growth rate in CGXII (0.34 h^−1^), the maximal biomass concentration was 53 and 12% higher in Lot1 and Lot2, respectively (8.9 g L^−1^, 6.5 g L^−1^, and 5.8 g L^−1^ CDW in Lot1, Lot2, and CGXII, respectively). In CGXII, 49 ± 2 mM L-lysine was produced, which was significantly lower than that obtained with the hydrolysates with 60 ± 3 mM and 55 ± 4 mM. Notably, in Lot2, 23 ± 1 mM L-glutamate was produced in addition, which was not the case in the hydrolysate of Lot1, underlining potential differences in various batches of sidestreams and their influence on production processes. Nevertheless, stable production could be demonstrated. Lot2 was chosen for further production experiments since more amino acids were available and as it contained higher concentrations of free xylose and arabinose (Supplementary Figure S3, Supplementary Table S2).

### Access to Lignocellulosic Sugars by Co-Overexpression of *xylA*_*Xc*_ *B*_*Cg*_ and *araBAD*_*Ec*_

**FIGURE 4:**
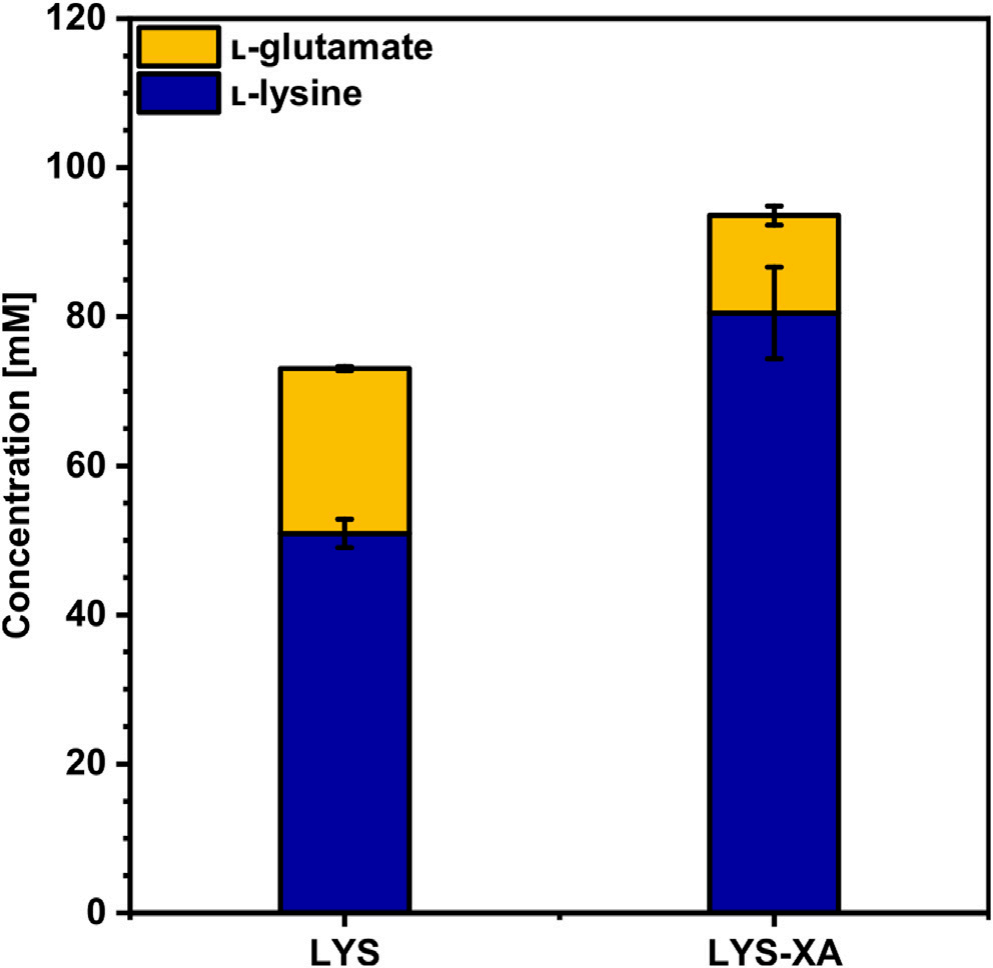
Production of l-lysine from WSCH by LYS and LYS-XA. *C. glutamicum* l-lysine producer strains LYS and LYS-XA were grown in the BioLector microcultivation system using WSCH, adjusted to 34 g L^−1^ glucose, and supplemented with 20 g L^−1^ ammonium sulfate and 42 g L^−1^ MOPS. Supernatants were analyzed after 48 h. Values and error bars represent means and standard deviations (n = of 3 cultivations). See Supplementary Table S3 for titers in g L^−1^.

To access the lignocellulosic pentoses arabinose and xylose in WSCH, heterologous expression of the genes *araBAD* from *Escherichia coli* (Kawaguchi et al., 2008; Schneider et al., 2011) and *xylA* from *Xanthomonas campestris* (Gopinath et al., 2011; Meiswinkel et al., 2013) as well as the overexpression of the native *xylB* (Buschke et al., 2013) under a synthetic constitutive strong promoter (Henke et al., 2021) on a single plasmid was established here. The resulting strain was called LYS-XA and clearly outcompeted its ancestor strain LYS. Consumption of 9 g L^−1^ xylose and 8 g L^−1^ arabinose on top of the 34 g L^−1^ (190 mM) glucose enabled the production of 81 ± 6 mM l-lysine and 13 ± 1 mM l-glutamate (Figure 4, Supplementary Table S3) by LYS-XA. Therefore, LYS-XA produced 56% more l-lysine than LYS (51 ± 2 mM) and grew up to 13% higher biomass concentration (Table 4), while about 41% less l-glutamate accumulated. Consequently, the product yield and volumetric productivity were significantly higher compared to those of LYS (Table 4), even though the growth was slightly inhibited by overexpressing *xylA*_*Xc*_ *B*_*Cg*_ *-araBAD*_*Ec*_ with growth rates of 0.23 ± 0.00 h^−1^ and 0.20 ± 0.00 h^−1^ for LYS and LYS-XA, respectively.

**TABLE 4:** Comparison of biomass formation (CDW), maximal growth rate (µ_max_), product yield (Y_P/X_), substrate yield (Y_P/S_), and volumetric productivity of l-lysine, cadaverine, and 5AVA producing *C. glutamicum* strains in CGXII minimal medium and WSCH.

Medium	Strain	CDW [g L^−1^]	µ_max_ [h-1]	Y_P/X_ [g g-1]	Y_P/S_ [g g^−1^]	VP [g L^−1^ h^−1^]
CGXII	LYS	5.8±0.3	0.31±0.00	1.24±0.10	0.18±0.01	0.42±0.02
	LYS-G	5.8±0.2	0.18±0.01	0.72±0.05	0.10±0.01	0.06±0.00
	CAD	5.7±0.3	0.27±0.00	1.08±0.07	0.15±0.01	0.19±0.01
	AVA1	6.0±0.3	0.14±0.00	1.20±0.04	0.18±0.00	0.24±0.01
	AVA2	6.9±0.0	0.20±0.00	0.37±0.03	0.06±0.00	0.13±0.01
	AVA1-G	4.9±0.4	0.05±0.00	0.96±0.03	0.12±0.01	0.05±0.00
	AVA2-G	6.8±0.2	0.07±0.00	0.58±0.03	0.08±0.00	0.06±0.00
WSCH	LYS	10.9±0.2	0.23±0.00	0.68±0.02	0.22±0.01	0.27±0.01
	LYS-G	10.5±0.4	0.19±0.00	0.60±0.04	0.16±0.01	0.21±0.01
	LYS-XA	12.3±0.4	0.20±0.00	0.95±0.05	0.24±0.02	0.37±0.03
	CAD	9.0±0.3	0.27±0.01	0.56±0.03	0.13±0.01	0.18±0.01
	CAD-XA	7.7±0.1	0.17±0.00	0.97±0.06	0.16±0.01	0.14±0.01
	AVA1	10.2±0.5	0.19±0.00	0.36±0.04	0.09±0.01	0.11±0.01
	AVA2	9.2±0.6	0.23±0.00	0.18±0.02	0.04±0.00	0.06±0.00
	AVA1-G	5.2±0.1	0.12±0.00	1.13±0.07	0.15±0.01	0.15±0.01
	AVA2-G	9.4±0.4	0.21±0.01	0.43±0.02	0.10±0.00	0.13±0.01

### Fermentative Production of Cadaverine From Wheat Sidestream Hydrolysates

**FIGURE 5:**
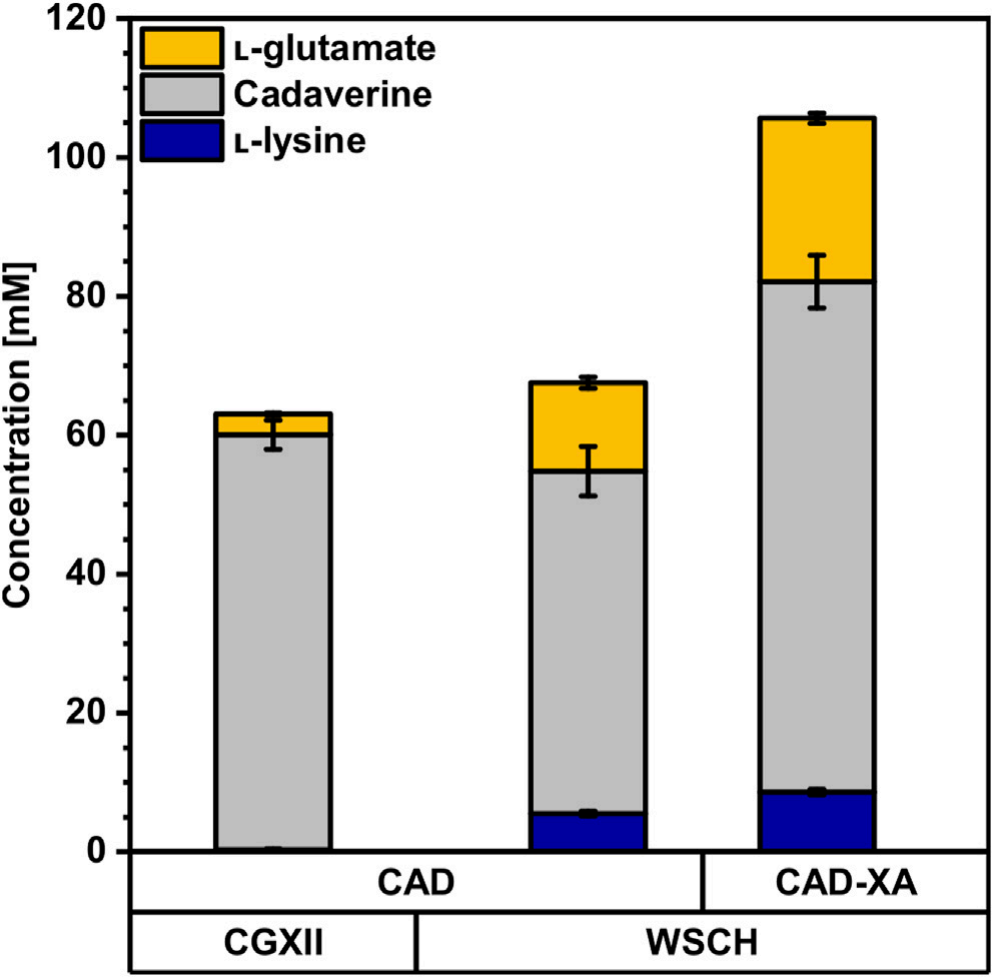
Production of cadaverine from CGXII minimal medium and the complex WSCH by CAD and CAD-XA. *C. glutamicum* cadaverine producer strains CAD and CAD-XA were grown in the BioLector microcultivation system using either CGXII minimal medium with 40 g L^−1^ glucose or in WSCH, adjusted to 34 g L^−1^ glucose, and supplemented with 20 g L^−1^ ammonium sulfate and 42 g L^−1^ MOPS. Both media were supplemented with 1 mM IPTG. Supernatants were analyzed after 72 h. Values and error bars represent means and standard deviations (n = of 3 cultivations). See Supplementary Table S3 for titers in g L^−1^.

Since WSCH supported l-lysine production by *C. glutamicum* well, the possibility to produce the l-lysine-derived C_5_-diamine cadaverine was investigated. The cadaverine overproducing *C. glutamicum* strain CAD heterologously expressed the gene coding for l-lysine decarboxylase (LdcC) from *E. coli*. In CGXII minimal medium with 40 g L^−1^ (220 mM) glucose CAD produced 60 ± 2 mM cadaverine, whereas cultivation in WSCH, adjusted to 34 g L^−1^ (190 mM) glucose, resulted in 49 ± 4 mM cadaverine (Figure 5, Supplementary Table S3). In contrast to the cultivation in CGXII, (i) less glucose was present as a carbon source, (ii) 6 ± 0 mM l-lysine accumulated and, (iii) 13 ± 1 mM l-glutamate and (iv) more biomass (Table 4) was formed. The growth rate remained stable at 0.27 h^−1^. The overexpression of *xylA*_*Xc*_ *B*_*Cg*_ and *araBAD*_*Ec*_ under a strong constitutive promoter in the cadaverine producer CAD-XA enabled access to xylose and arabinose as additional carbon sources. Strain CAD-XA consumed 8 g L^−1^ xylose and 6 g L^−1^ arabinose, and the cadaverine titer was increased by 50% (74 ± 4 mM); 56% more l-lysine was formed (9 ± 0 mM), and the l-glutamate titer almost doubled (24 ± 1 mM).

### Comparison of Two Different Routes for 5AVA Production From Wheat Sidestream Hydrolysates

**FIGURE 6:**
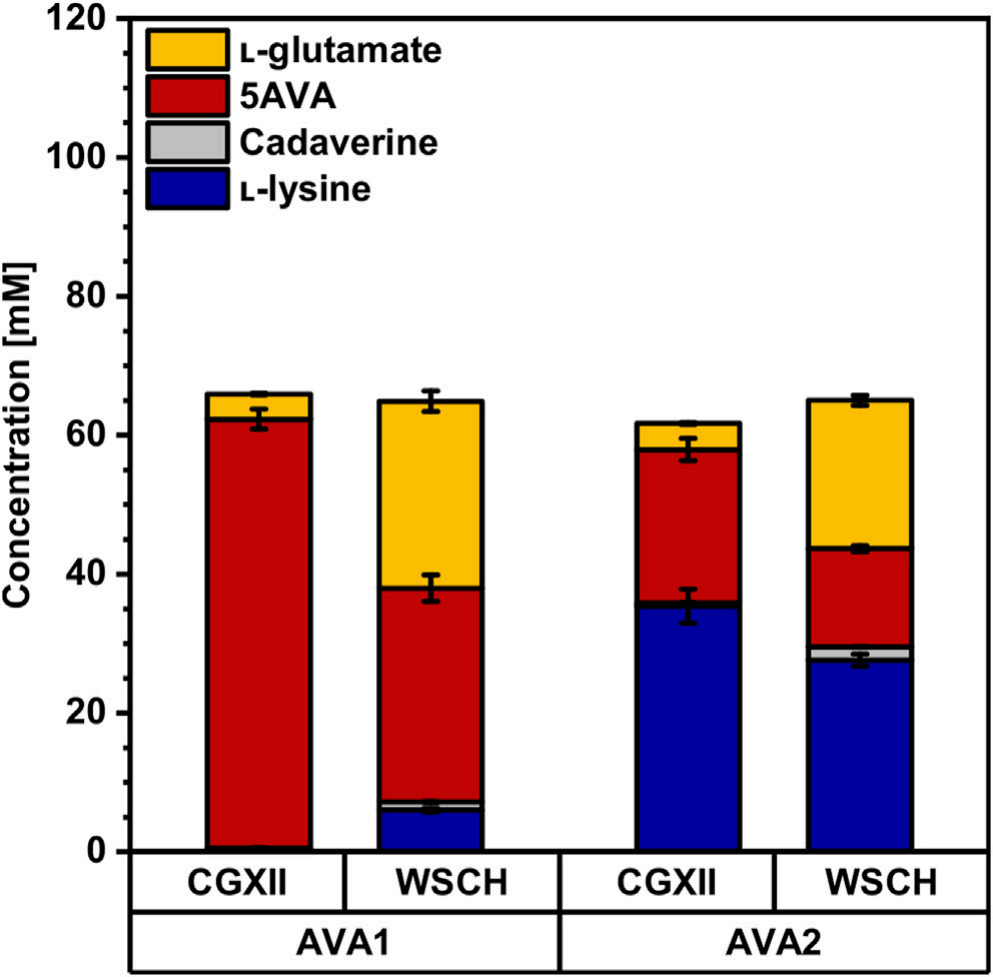
Production of 5AVA from CGXII minimal medium and the complex WSCH by AVA1 and AVA2. *C. glutamicum* 5AVA producer strains AVA1 and AVA2 were grown in the BioLector microcultivation system using either CGXII minimal medium with 40 g L^−1^ glucose or in WSCH, adjusted to 34 g L^−1^ glucose, and supplemented with 20 g L^−1^ ammonium sulfate and 42 g L^−1^ MOPS. Both media were supplemented with 1 mM IPTG. Supernatants were analyzed after 48 h. Values and error bars represent means and standard deviations (n = of 3 cultivations). See Supplementary Table S3 for titers in g L^−1^.

As cadaverine production from WSCH was established, the pathway was further extended for the production of the C_5_-ω-amino acid 5AVA. Therefore, two different routes were tested. First, using the route LdcC-PatA-PatD (=AVA1) (Jorge et al., 2017b), 62 ± 1 mM 5AVA was produced in minimal medium without the accumulation of the precursors l-lysine or cadaverine in CGXII minimal medium (Figure 6, Supplementary Table S3). The second route LdcC-Puo-PatD (=AVA2) that requires molecular oxygen (Haupka et al., 2020) only yielded 22 ± 2 mM 5AVA, while 35 ± 2 mM L-lysine accumulated as a by-product. Even though AVA2 showed incomplete conversion of l-lysine to 5AVA, one advantage of this strain was its higher growth rate (0.20 ± 0.00 h^−1^ compared to 0.14 ± 0.00 h^−1^; Table 4).

Using WSCH, 5AVA production decreased significantly. AVA1 produced only 50% 5AVA (31 ± 2 mM) and accumulated 1 ± 0 mM cadaverine and 6 ± 0 mM l-lysine as by-products. Notably, up to 27 ± 2 mM l-glutamate was formed, whereas only 4 ± 0 mM was produced in minimal medium. However, growth was accelerated in WSCH by 36% (0.19 ± 0.00 h^−1^), and more biomass was formed (Table 4, CGXII: 6.0 ± 0.3 g L^−1^, WSCH: 10.2 ± 0.5 g L^−1^). Growth and production of AVA2 in WSCH was affected in a similar way as AVA1. While the total production was decreased (14 ± 1 mM 5AVA, 2 ± 0 mM cadaverine, and 28 ± 1 mM l-lysine), more l-glutamate was formed as by-products (21 ± 1 mM) than in minimal medium (4 ± 0 mM). Also, 33% more biomass was formed, and the maximal growth rate increased up to 0.23 ± 0.00 h^−1^ (Table 4).

### Deletion of *gdh* for Improved 5AVA Production

**FIGURE 7:**
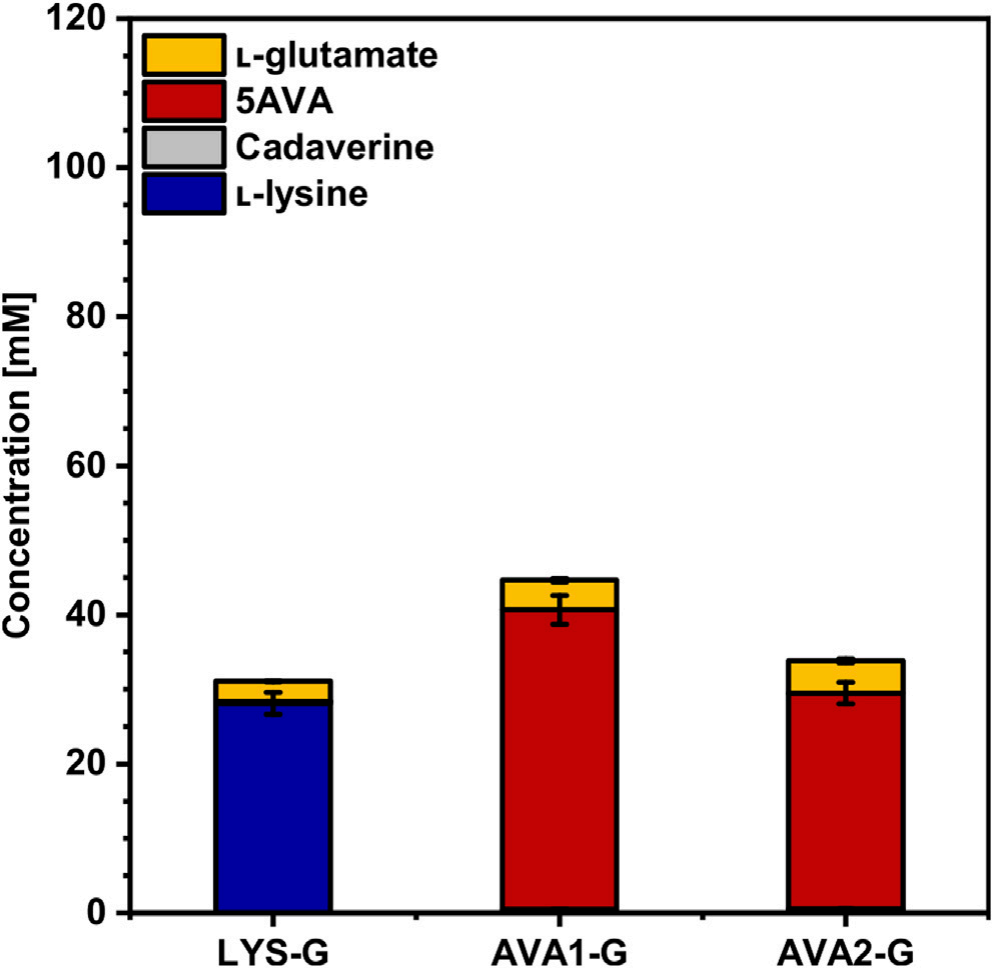
Effect of *gdh* deletion on the production of l-lysine and 5AVA from CGXII minimal medium by *C. glutamicum*. The *gdh*-deficient strains LYS-G, AVA1-G, and AVA2-G were grown in the BioLector microcultivation system using CGXII minimal medium with 40 g L^−1^ glucose, supplemented with 1 mM IPTG. Supernatants were analyzed after 120 h. Values and error bars represent means and standard deviations (n = of 3 cultivations). See Supplementary Table S3 for titers in g L^−1^.

As l-glutamate turned out to be a main by-product in WSCH cultivations, the gene *gdh*, coding for l-glutamate dehydrogenase, was deleted in LYS, AVA1 and AVA2, resulting in the strains LYS-G, AVA1-G and AVA2-G. As Gdh is the main nitrogen assimilating enzyme, growth in CGXII minimal medium and l-lysine production are severely affected (Prell et al., 2021b), but can be restored by flux enforcement over one (Haupka et al., 2020) or two (Pérez-García et al., 2018) coupling site(s). First, the impact of the coupling site over the putrescine transaminase PatA in AVA1-G compared to that in AVA2-G (no coupling site) was investigated in CGXII minimal medium. It was demonstrated that one coupling site was superior to no coupling site (Figure 7, Supplementary Table S3). AVA1-G (with the coupling site) still produced 40 ± 2 mM 5AVA (-35% compared to AVA1, Figure 6) with no precursor accumulation, even though l-lysine production dropped by 42% from 49 ± 2 mM (LYS, Figure 3) to 28 ± 2 mM (LYS-G, Figure 7). AVA2-G (no coupling site) produced with a titer of 29 ± 2 mM 28% less 5AVA than AVA1-G, but upon deletion of *gdh*, no accumulation of l-lysine could be observed. Moreover, the maximal growth rate of all three *gdh*-deficient strains decreased (Table 4). Additionally, AVA1-G displayed with one coupling site, higher substrate and product yields (Y_P/S_: 0.12 ± 0.01 g g^−1^, Y_P/X_ 0.96 ± 0.03 g g^−1^) compared to AVA2-G (Y_P/S_: 0.08 ± 0.00 g g^−1^, Y_P/X_: 0.58 ± 0.03 g g^−1^) with no coupling site.

**FIGURE 8:**
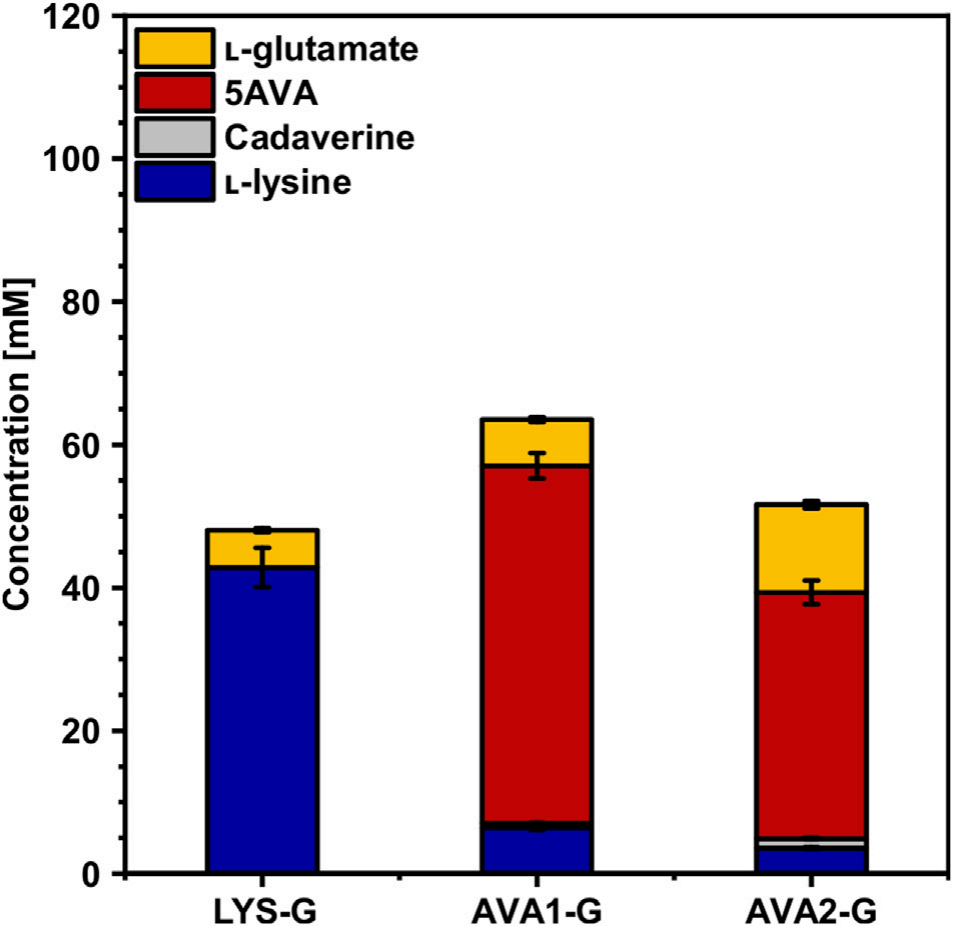
Effect of *gdh* deletion on the production of l-lysine and 5AVA from WSCH by *C. glutamicum*. The *gdh*-deficient strains LYS-G, AVA1-G and AVA2-G were grown in the BioLector microcultivation system in WSCH, adjusted to 34 g L^−1^ glucose, supplemented with 20 g L^−1^ ammonium sulfate and 42 g L^− 1^ MOPS, and induced with 1 mM IPTG. Supernatants were analyzed after 48 h. Values and error bars represent means and standard deviations (n = of 3 cultivations). See Supplementary Table S3 for titers in g L^−1^.

Since cultivation in WSCH accelerated the growth of AVA1 and AVA2, the *gdh*-deficient strains AVA1-G and AVA2-G were tested in WSCH. Indeed, the growth of AVA1-G and AVA2-G was improved significantly compared to that of CGXII minimal medium. The maximal growth rate improved 2.4-fold for AVA1-G and 3.0-fold for AVA2-G (Table 4). Furthermore, 24% (AVA1-G) and 19% (AVA2-G) more 5AVA was produced in WSCH compared to that in CGXII, increasing volumetric productivity 3-fold and 2-fold, respectively (Table 4). The *gdh*-deficient strains AVA1-G and AVA2-G additionally surpassed their parental strains AVA1 and AVA2 cultivated in WSCH in terms of production (Figures 6, 8, Supplementary Table S3), substrate yield, and product yield (Table 4). As expected, the deletion of *gdh* led to lower l-glutamate titers (AVA1-G: 7 ± 0 mM, AVA2-G: 12 ± 1 mM), and in return, more amount of product was formed. With a concentration of 34 ± 2 mM 5AVA and only 4 ± 0 mM L-lysine as the by-product, AVA2-G, cultivated in WSCH, outcompeted its parental strain in CGXII minimal medium and WSCH. AVA1-G (Figure 6) produced up to 50 ± 2 mM 5AVA (62% more compared to AVA1), and thus, it was the best 5AVA producer in WSCH medium. Moreover, it could be demonstrated that the deletion of *gdh* resulted in decreased l-glutamate titers (5 ± 3 mM) in the *gdh*-deficient l-lysine producer LYS-G (Figure 8) compared to that of its parental strain LYS (13 ± 1 mM, Figure 4). In contrast to the 5AVA producers, the growth rate was barely improved (0.19 ± 0.00 h^−1^) in comparison to cultivation in CGXII minimal medium (0.18 ± 0.01 h^−1^), and the l-lysine concentration also decreased (43 ± 3 mM), i.e., the effect of deleting *gdh* was weakened by the cultivation in WSCH, but not cured.

## Discussion

In this study, WSC from the paper industry was shown to be a sustainable feedstock for the fermentative production of the amino acid l-lysine, the diamine cadaverine, and the ω-amino acid 5AVA. Hydrolysis of the sidestream and enabling access to pentose utilization improved production. Deletion of *gdh* reduced formation of the by-product glutamate and coupled growth to production by flux enforcement. Strain AVA1-G produced 5.9 ± 0.2 g L^−1^ 5AVA from WSCH.

Utilization of hydrolysates from alternative feedstocks has been demonstrated for *C. glutamicum* before for products such as l-glutamate and l-lysine (Gopinath et al., 2011), *N*-ethylglycine (Mindt et al., 2019a), and succinate (Mao et al., 2018). Similar to those studies, among the tested hydrolysates of WSC with acid, base, and water, it was found to be most efficient with 4% (*w/v*) H_2_SO_4_ as judged from the yield of glucose, xylose, and arabinose. During acidic hydrolysis, starch and maltose are degraded to glucose (BeMiller and Mann, 1966; Abbadi et al., 1998), which explains the high glucose concentration in WSCH_H2SO4_. Hydrolysis of cellulose and hemicellulose from lignocellulose could have led to the release of glucose, xylose, and arabinose (Debiagi et al., 2020). Typically, lignocellulosic hydrolysates contain growth inhibitors like furfural, hydroxymethylfurfural, formic acid, and phenolic compounds (Palmqvist and Hahn-Hägerdal, 2000), to which *C. glutamicum* possesses moderate tolerance, and in growth-arrested *C. glutamicum* R cells, productivity has been shown to be little or not affected in the presence of various inhibitors (Sakai et al., 2007). In this study, a growth-inhibiting effect of high WSCH concentrations was observed (data not shown), likely due to sugar-derived inhibitors resulting from hydrolysis of lignocellulose. In order to increase WSCH concentrations, reduce lag phases, and reach higher growth rates, hydrolysates would need to be detoxified. Many approaches for detoxification of hydrolysates have been developed. In *C. glutamicum* S9114, several oxidoreductases were identified which helped to increase growth and inhibitor conversion. Overexpression of CGS9114_RS01115 led to increased conversion of five inhibitory aldehydes including furfural, hydroxymethylfurfural, 4-hydroxybenzaldehyde, vanillin, and syringaldehyde (Zhou et al., 2019). Koopman et al. identified the furfural and hydroxymethylfurfural degradation pathways of *Cupriavidus basilensis* HMF14 and enabled *Pseudomonas putida* to detoxify the aldehydes *in situ* and utilize them as a carbon source by degrading them to 2-oxoglutarate (Koopman et al., 2010). In contrast to targeted gene expression for *in situ* detoxification, several studies reported increased tolerance to inhibitors in lignocellulosic hydrolysates by UV mutagenesis of *Scheffersomyces shehatae* (Senatham et al., 2016) or adaptive laboratory evolution as shown for *C. glutamicum* S9114 in corn stover hydrolysates (Wang et al., 2018). However, these approaches should be accompanied by whole genome re-sequencing to exclude mutational changes at the expense of production. As an alternative to increase tolerance against inhibitors in hydrolysates, hydrolysates can be prepared or treated enzymatically to reduce the accumulation of inhibitors. Treatment with laccase and lignin peroxidase removed phenolic compounds almost completely (Chen et al., 2019). Preparation of hydrolysates with the cellulolytic enzyme reagent Cellic^®^ Ctec2 instead of acidic hydrolysis prevented the accumulation of inhibitors (Mao et al., 2018). Detoxified WSCH could improve the growth of production strains and ultimately, productivity, without the need for heterologous expression of additional genes or adaptive laboratory evolution.

As the chemical composition in wheat and wheat hydrolysates varies in different years and batches (Rosicka-Kaczmarek et al., 2013), hydrolysates from two batches were compared in this study (Figure 3). WSCH Lot2 contained more xylose and arabinose than Lot1, which could be utilized for enhanced l-lysine and cadaverine production by the expression of the genes *xylA*_*Xc*_ *B*_*Cg*_ and *araBAD*_*Ec*_. Apart from sugars, hydrolysates may contain aromatic compounds, which can be consumed by *C. glutamicum*, e.g., benzoate, phenol, 3-hydroxybenzoate, protocatechuate, vanillate, benzyl alcohol, and ferulate (Shen et al., 2012). Strikingly, the strain LYS not only produced 55 ± 4 mM l-lysine in WSCH Lot2, but also additional 23 ± 1 mM l-glutamate, while producing almost no l-glutamate in Lot1. This cannot be attributed to a difference in the initial l-glutamate concentrations in Lot1 (5 mM) and Lot2 (6 mM) and, therefore, must be due to regulatory effects. In *C. glutamicum,* l-glutamate production is triggered, i.a., by biotin limitation (Eggeling and Sahm, 1999). It has been reported that biotin availability in different batches of wheat varies and that the content of free biotin and its bioavailability are much lower in wheat compared to that in corn, barley, and oats (Frigg, 1976; Anderson et al., 1978; Bryden et al., 1991). Thus, biotin limitation might play a role here, other than in the biotin-rich corn stover hydrolysate, for which *C. glutamicum* S9114 had to be metabolically engineered in order to overcome inhibition by biotin and to produce l-glutamate (Wen and Bao, 2019). The key enzyme to regulate l-glutamate overproduction, the 2-oxoglutarate dehydrogenase complex (ODHC), is the target of phosphorylation by the inhibitor OdhI, and OdhI is phosphorylated by PknG. On the one hand, WSCH Lot2 might contain inhibitors of ODHC like *cis*-aconitate, oxalacetate, and pyruvate (Shiio and Ujigawa-Takeda, 1980), and potentially, OdhI and PknG might be influenced by effectors in WSCH as well with the effect of ODHC repression, leading to increased l-glutamate production. On the other hand, under l-glutamate producing conditions, the global posttranslational protein acetylation in *C. glutamicum* decreases and succinylation increases, affecting proteins in carbohydrate metabolism, translation, and amino acid metabolism (Mizuno et al., 2016). If perhaps increased succinylation status is triggered in WSCH Lot2 compared to that in Lot1, this might lead to increased l-glutamate production.

When the l-lysine pathway was extended to cadaverine and 5AVA, incomplete conversion of l-lysine was observed in WSCH (Figures 5, 6, 8), making the decarboxylation of l-lysine *via* LdcC the limiting step in the pathway. Since this was not observed in minimal medium, either LdcC is inhibited by an unknown inhibitor in WSCH or its cofactor pyridoxal 5′-phosphate (PLP) is limiting. PLP and l-glutamate are synthesized from d-ribose 5-phosphate, d-glyceraldehyde 3-phosphate, and l-glutamine *via* the PLP synthase complex PdxST (Jochmann et al., 2011). Since l-glutamate accumulates in WSCH, l-glutamine synthesis *via* glutamine synthetase GlnA might be hampered, resulting in low l-glutamine content and, consequently, low PLP synthesis. Whether PLP limitation, in fact, is the cause for incomplete l-lysine decarboxylation could be investigated by PLP addition to the WSCH medium. In *E. coli* whole-cell biocatalysis, supplementation of PLP or heterologous expression of *pdxS* and *pdxT* from *Bacillus subtilis* increased cadaverine productivity by 2.9 folds (Ma et al., 2015).

Flux enforcement can be used as a means to couple production to another condition to increase the flux through the targeted pathway. By deletion of the l-glutamate dehydrogenase–encoding gene *gdh*, synthesis of l-glutamate is impaired, providing the opportunity to couple growth to production of 5AVA *via* 2-oxoglutarate-dependent putrescine/cadaverine transaminase PatA. Haupka et al. have shown that flux enforcement is more effective with one coupling site than with two (Haupka et al., 2020). Deletion of *gdh* in the strain LYS-G resulted in 42% decreased l-lysine production (28 ± 2 mM) in minimal medium (Figure 7) due to a missing coupling site to synthesize l-glutamate. Prell et al. showed that nitrogen starvation was partially triggered by *gdh* deletion, leaving the GS/GOGAT system as a l-glutamate replenisher with an increased energy demand compared to Gdh, which resulted in decreased growth rates in glutarate-producing strains (Prell et al., 2021b). In AVA1-G, however, which possesses a coupling site for l-glutamate synthesis *via* PatA, 40 ± 2 mM 5AVA was produced, thereby exceeding the l-lysine production in LYS-G, which hints at the effectiveness of flux enforcement. This is in line with the lower 5AVA production in AVA2-G, which has no coupling site due to use of the putrescine oxidase gene *puo*. In WSCH, the production of l-lysine and 5AVA by the strains LYS-G, AVA1-G, and AVA2-G exceeded production in minimal medium (Figures 7, 8) with AVA1-G as the highest 5AVA producer in WSCH (50 ± 2 mM). Even without the flux enforcement, the deletion of *gdh* improved production in WSCH as 2-oxoglutarate is not converted to l-glutamate, but further driven through the TCA cycle toward oxaloacetate, the precursor for l-lysine biosynthesis. Since WSCH is rich in amino acids including l-glutamate, the nitrogen starvation response, regulated by AmtR (Schulz et al., 2001), might have been canceled despite of *gdh* deletion in these strains, resulting in an escape of the metabolic deficit that is accompanied by deletion of *gdh* and the nitrogen starvation response. In a glutarate-producing *C. glutamicum* strain carrying a *gdh* deletion, which was evolved by adaptive laboratory evolution, RNAseq analysis revealed that many genes of the AmtR regulon were downregulated in comparison with the unevolved strain due to the amino acid exchange E686Q in the large subunit GltB of GOGAT (Prell et al., 2021b). The mechanism for nitrogen sensing in *C. glutamicum* is not known yet, but it is conceivable that the nitrogen starvation response in AVA1-G is downregulated in WSCH compared to that in minimal medium as it is for the evolved glutarate producer.

The fundamental establishment of WSCH as a medium for production of 5AVA in *C. glutamicum* was shown here; however, a scale-up approach is necessary in order to reach industrial relevance. *C. glutamicum* has been employed for bioreactor fermentation with several hydrolysates. Rice straw hydrolysate was used as a carbon source in CGXII medium for the production of 1.6 g L^−1^ *N*-ethylglycine in a fed-batch cultivation with a 2 L starting volume (Mindt et al., 2019a). *Miscanthus* hydrolysate was utilized as a carbon source in CG100 medium for the production of 12.5 g L^−1^ 5AVA in a 500 mL batch cultivation (Joo et al., 2017). For the production of succinate in 3.5 L batch fermentation, *C. glutamicum* cells were immobilized in the bioreactor using a porous polyurethane filler, and *cassava bagasse* hydrolysate was used yielding 22.5 g L^−1^ succinate (Shi et al., 2014). WSCH can substitute most of the components of CGXII minimal medium; therefore it is likely that for upscaling of 5AVA production, a similar mixture of WSCH and ammonium sulfate can be utilized as it has been used here, with the exception of MOPS buffer, since pH is regulated automatically during bioreactor fermentation.

Future applications of WSCH may target glutarate (Pérez-García et al., 2018) and l-2-hydroxyglutarate (Prell et al., 2021a) since the pathway only needs to be extended by additional two and three reactions, respectively. Both compounds were produced from unhydrolyzed WSC (Prell et al., 2021a). Furthermore, reactive extraction has been proven to be an effective method for downstream processing of glutarate from CGXII fermentation broth (Prell et al., 2021b). In future, it has to be studied if the developed reactive extraction/re-extraction methods can be applied to WSCH-based production processes.

## Data Availability

The original contributions presented in the study are included in the article/Supplementary Material. Further inquiries can be directed to the corresponding author.
