# Supplementary material for: Utilization of a Wheat Sidestream for 5-Aminovalerate Production in Corynebacterium glutamicum
Source: Front Bioeng Biotechnol. 2021 Sep 29;9:732271. doi: 10.3389/fbioe.2021.732271 (PMC8511785; doi:10.3389/fbioe.2021.732271)
Supplement: Supplementary file 1 [file Table1.DOCX]

Supplementary Material

**Figure S1:** Chromatograms of the amino acid analysis of the different hydrolysates (H_2_O: blue; H_2_SO_4_: red, NaOH: green) in comparison to the wheat sidestream concentrate (black).

**Table S1: Heat map of amino acid concentrations in the wheat sidestream concentrate (WSC) and its hydrolysates. Concentrations are given in mM/100g dry weight.**

| **Amino acid** | **WSC** | **WSCH_H2O_** | **WSCH_NaOH_** | **WSCH_H2SO4_** |
| --- | --- | --- | --- | --- |
| l-aspartate | **0.4** | **0.9** | **2.2** | **3.9** |
| l-glutamate | **0.4** | **0.4** | **4.8** | **6.0** |
| l-serine | **0.1** | **0.2** | **0.6** | **1.2** |
| l-glutamine | **0.1** | **0.1** | **0.5** | **0.1** |
| l-histidine | **0.1** | **0.0** | **0.0** | **0.0** |
| l-glycine | **0.0** | **0.4** | **3.3** | **2.9** |
| l-threonine | **0.2** | **0.5** | **4.5** | **3.5** |
| l-tyrosine/ l-alanine/ l-arginine | **0.1** | **0.3** | **1.7** | **1.5** |
| l-tryptophane | **0.0** | **0.1** | **0.3** | **0.1** |
| l-methionine | **0.1** | **0.0** | **0.0** | **0.1** |
| l-valine | **0.1** | **0.2** | **0.8** | **1.1** |
| l-phenylalanine | **0.0** | **0.2** | **0.6** | **0.7** |
| Isoleucine | **0.1** | **0.1** | **0.4** | **0.6** |
| l-leucine | **0.2** | **0.4** | **1.4** | **2.0** |
| l-lysine | **0.3** | **0.9** | **1.1** | **1.7** |

**Figure S2:** Chromatograms of the carbohydrate analysis of the different hydrolysates (H_2_O: blue; H_2_SO_4_: red, NaOH: green) in comparison to the wheat sidestream concentrate (black).

**Figure S3:** Chromatograms for comparison of the amino acid (A-B) and the carbohydrates (C-D) analysis of Lot1 (black) and Lot2 (red).

**Table S2: Comparison of amino acid content in the acidic hydrolysates from Lot1 and Lot2. Concentrations are given in mM/100 g dry weight.**

|  | **Lot 1** | **Lot 2** |
| --- | --- | --- |
| **Amino acids** |  |  |
| l-aspartate | **3.4** | **4.8** |
| l-glutamate | **5.6** | **4.2** |
| l-serine | **1.1** | **1.3** |
| l-glutamine | **0.1** | **0.1** |
| l-histidine | **0.9** | **0.9** |
| l-glycine | **2.9** | **3.5** |
| l-threonine | **3.7** | **4.7** |
| l-tyrosine/ l-alanine/ l-arginine | **1.4** | **1.7** |
| l-tryptophane | **0.3** | **0.4** |
| l-methionine | **0.2** | **0.3** |
| l-valine | **1.1** | **1.2** |
| l-phenylalanine | **0.6** | **0.8** |
| Isoleucine | **0.6** | **0.6** |
| l-leucine | **1.8** | **2.0** |
| l-lysine | **1.6** | **2.1** |
| **Carbohydrates** |  |  |
| Maltose | **7.3** | **10.0** |
| Glucose | **164.1** | **157.0** |
| Xylose | **34.1** | **62.3** |
| Arabinose | **18.4** | **34.7** |
| Acetate | **0.0** | **22.3** |

**Table S3: Titers of l-lysine, cadaverine, 5AVA and l-glutamate from figures 3 – 8 in g L^-1^. WSCH Lot 2 was used and adjusted to 34 g L^-1^ (190 mM) glucose, if not stated otherwise.**

| **Medium** | **Strain** | **l-lysine [g L^-1^]** | **Cadaverine [g L^-1^]** | **5AVA [g L^-1^]** | **l-glutamate [g L^-1^]** |
| --- | --- | --- | --- | --- | --- |
| CGXII | LYS | 7.1 ± 0.4 | - | - | 0.4 ± 0.0 |
| WSCH Lot 1 (40 g L^-1^ glucose) | LYS | 8.9 ± 0.5 | - | - | 0.1 ± 0.0 |
| WSCH Lot 2 (40 g L^-1^ glucose) | LYS | 8.3 ± 0.6 | - | - | 3.3 ± 0.1 |
| WSCH | LYS | 7.4 ± 0.3 | - | - | 3.2 ± 0.0 |
| WSCH | LYS-XA | 11.8 ± 1.1 | - | - | 1.9 ± 0.2 |
| CGXII | CAD | 0.0 ± 0.0 | 6.1 ± 0.3 | - | 0.4 ± 0.0 |
| WSCH | CAD | 0.8 ± 0.1 | 5.0 ± 0.4 | - | 1.9 ± 0.1 |
| WSCH | CAD-XA | 1.3 ± 0.1 | 7.5 ± 0.5 | - | 3.4 ± 0.1 |
| CGXII | AVA1 | 0.0 ± 0.0 | 0.0 ± 0.0 | 7.2 ± 0.2 | 0.5 ± 0.0 |
| WSCH | AVA1 | 0.9 ± 0.0 | 0.1 ± 0.0 | 3.6 ± 0.3 | 3.9 ± 0.2 |
| CGXII | AVA2 | 5.2 ± 0.4 | 0.1 ± 0.0 | 2.6 ± 0.2 | 0.5 ± 0.0 |
| WSCH | AVA2 | 4.0 ± 0.2 | 0.2 ± 0.0 | 1.7 ± 0.1 | 3.1 ± 0.1 |
| CGXII | LYS-G | 4.1 ± 0.3 | - | - | 0.4 ± 0.0 |
| CGXII | AVA1-G | 0.0 ± 0.0 | 0.0 ± 0.0 | 4.7 ± 0.3 | 0.6 ± 0.0 |
| CGXII | AVA2-G | 0.0 ± 0.0 | 0.0 ± 0.0 | 3.4 ± 0.2 | 0.6 ± 0.0 |
| WSCH | LYS-G | 6.3 ± 0.5 | - | - | 0.8 ± 0.0 |
| WSCH | AVA1-G | 0.9 ± 0.0 | 0.1 ± 0.0 | 5.9 ± 0.3 | 0.9 ± 0.0 |
| WSCH | AVA2-G | 0.5 ± 0.0 | 0.1 ± 0.0 | 4.0 ± 0.2 | 1.8 ± 0.01 |
